# Evidence-informed policies: tools for decision-making in the Brazilian Unified Health System

**DOI:** 10.1590/S2237-96222026v35e20263005.en

**Published:** 2026-09-28

**Authors:** Carolina Müller Ferreira, Maria Auxiliadora Parreiras Martins, Everton Nunes da Silva, Jorge Otávio Maia Barreto, Taís Freire Galvão

**Affiliations:** 1 Universidade de Brasília, Programa de Pós-Graduação em Ciências Farmacêuticas, Brasília, DF, Brazil; 2 Universidade Federal de Minas Gerais, Faculdade de Farmácia, Belo Horizonte, MG, Brazil; 3 Universidade de Brasília, Faculdade de Ciências e Tecnologias em Saúde, Brasília, DF, Brazil; 4 Fundação Oswaldo Cruz, Gerência Regional de Brasília, Brasília, DF, Brazil; 5 Universidade Estadual de Campinas, Faculdade de Ciências Farmacêuticas, Campinas, SP, Brazil

Decision-making and public health policy implementation require assessment of benefits, risks, costs, equity and feasibility supported by scientific evidence^1^. Evidence networks, comprising researchers and policymakers, assist governments and institutions in utilizing reliable data for decision-making through capacity building, production of knowledge syntheses, communication, technical-scientific support and public health monitoring^2^. This form of collaboration between researchers and decision-makers fosters the formulation of research questions that are better aligned with the real-world challenges faced by the population and by health service and health system management. As a journal of the Brazilian Unified Health System (*Sistema Único de Saúde*, SUS), *Epidemiologia e Serviços de Saúde: revista do SUS* (RESS) prioritizes dissemination of evidence relevant to decision-making^3^, such as highlighting systematic reviews and evidence syntheses that facilitate knowledge about the effects of strategic interventions on SUS care^4^ ^-^ ^7^.

In 2007, the World Health Organization launched the Evidence-informed Policy Network (EVIPNet) as a global initiative to strengthen the capacity of countries to use scientific evidence in the formulation, implementation and evaluation of public health policies^8^. EVIPNet supports institutionalization of evidence-informed policies by fostering collaboration between researchers, managers and policymakers, promoting capacity building, production of evidence syntheses, deliberative dialogues, knowledge translation and technical-scientific advice to inform health decisions. In Brazil, EVIPNet is coordinated by the Brazilian Ministry of Health through its Department of Science and Technology, part of its Health Science, Technology and Innovation Secretariat^8^.

With the aim of reaffirming its commitment to strengthening and disseminating evidence, and in partnership with EVIPNet Brazil, RESS launched a special call for papers on evidence-informed policies research for the second half of 2025. The initiative sought to gather and disseminate original articles and reviews produced by researchers on topics of interest for public health. These studies highlighted evidence synthesis methods common to the field of evidence-informed policy; consequently, six reviews and three original articles have been published. These works provide a basis for public health policy decision-making and form the subject of this editorial.

A survey conducted between 2024 and 2025 regarding Brazil's Evidence Centers (*Núcleos de Evidência*, NEv) found that, despite the availability of basic infrastructure and access to scientific databases, limitations persist due to a scarcity of financial resources and qualified personnel^9^. Similarly, an assessment of the institutional capacity of the five departments within the Health Science, Technology and Innovation Secretariat in 2024 identified highly qualified professionals and knowledge regarding evidence-informed policy, but also revealed disparities between departments and barriers to evidence use, such as time constraints, difficulties accessing scientific articles and lack of knowledge translation tools^10^. Additionally, a scoping review demonstrated that health residency programs contribute to strengthening human capital, enhancing professional practice and fostering integration of education and service delivery in Brazil^11^.

An evidence synthesis presented strategies for addressing vaccine hesitancy and identified options such as strengthening communication, implementing education programs for health professionals, expanding public access to vaccination and integrating vaccination strategies for pregnant women^12^. A rapid review of systematic reviews showed that use of insecticides, community reporting, monitoring of domestic and wild animals, as well as educational and management measures, would be important for controlling and preventing Chagas disease^13^. These syntheses can support the implementation of programs and actions within the context of the SUS.

Another review of systematic reviews examined sexual and reproductive health promotion among adolescents and young adults in Brazil. It found that most interventions took place in school settings, focusing on contraceptive use and highlighting the importance of adopting a multidisciplinary approach to health promotion^14^. A cross-sectional study involving schoolchildren in the city of Cubatão indicated that exposure to air pollution was associated with reduced heart rate variability and lung function^15^.

The influence of inequalities on aspects of care and evidence generation was discussed in several articles. A scoping review revealed that while most studies explored the biological nature of impairments, few addressed sociopolitical markers, highlighting gaps in how this issue is addressed in Brazil^16^. A systematic review identified that COVID-19 hospitalizations and mortality in Brazil were likely driven by socioeconomic inequalities and unequal access to healthcare, with a disproportionate impact on Black individuals^17^. These contributions take the Brazilian context into account and support the implementation of evidence-informed policies in the areas analyzed.

The studies submitted in response to the RESS call for papers, prompted by the EVIPNet Brazil Network, synthesized global and local evidence for informing public health policy decision-making. The inclusion of diverse topics and methods demonstrates the maturity of the research fostered within the EVIPNet Brazil context in providing evidence for decision-making. By publishing these research findings, RESS reaffirms its role in disseminating evidence that contributes to the enhancement of the SUS.
