# Supplementary material for: Evidence-informed policies: tools for decision-making in the Brazilian Unified Health System
Source: Epidemiol Serv Saude. 2026 Sep 28;35:e20263005. doi: 10.1590/S2237-96222026v35e20263005.en (PMC13626268; doi:10.1590/S2237-96222026v35e20263005.en)
Supplement: Translation

# Translation: Políticas informadas por evidências: ferramentas para a tomada de decisão no Sistema Único de Saúde

**DOI:** 10.1590/S2237-96222026v35e20263005.pt

**Authors:** Carolina Müller Ferreira, Maria Auxiliadora Parreiras Martins, Everton Nunes da Silva, Jorge Otávio Maia Barreto, Taís Freire Galvão

**Affiliations:** 1 Universidade de Brasília, Programa de Pós-Graduação em Ciências Farmacêuticas, Brasília, DF, Brasil; 2 Universidade Federal de Minas Gerais, Faculdade de Farmácia, Belo Horizonte, MG, Brasil; 3 Universidade de Brasília, Faculdade de Ciências e Tecnologias em Saúde, Brasília, DF, Brasil; 4 Fundação Oswaldo Cruz, Gerência Regional de Brasília, Brasília, DF, Brasil; 5 Universidade Estadual de Campinas, Faculdade de Ciências Farmacêuticas, Campinas, SP, Brasil

A tomada de decisão e a implementação de políticas públicas em saúde requerem que benefícios, riscos, custos, equidade e viabilidade sejam avaliados com base em evidências científicas^1^. As redes de evidências, compostas por pesquisadores e formuladores de políticas públicas, auxiliam governos e instituições a usar dados confiáveis na tomada de decisão por meio de capacitações, produções de síntese de conhecimento, comunicação, apoio técnico-científico e monitoramento da saúde pública^2^. Essa aproximação entre pesquisadores e tomadores de decisão estimula a definição de perguntas de pesquisa mais alinhadas aos problemas reais vivenciados pela população e enfrentados pela gestão dos serviços e sistemas de saúde. Como periódico do Sistema Único de Saúde (SUS), a *Epidemiologia e Serviços de Saúde: revista do SUS* (RESS) tem como prioridade disseminar evidências relevantes para a tomada de decisão^3^, com destaque para revisões sistemáticas e sínteses de evidências que facilitam o conhecimento sobre os efeitos de intervenções estratégicas para a assistência no SUS^4^ ^-^ ^7^.

Em 2007, a Organização Mundial da Saúde lançou a *Evidence-informed Policy Network* (EVIPNet) como uma iniciativa global para fortalecer a capacidade dos países de utilizar evidências científicas na formulação, implementação e avaliação de políticas públicas de saúde^8^. A EVIPNet apoia a institucionalização de políticas informadas por evidências mediante a articulação entre pesquisadores, gestores e formuladores de políticas, promovendo o desenvolvimento de capacidades, a produção de sínteses de evidências, a realização de diálogos deliberativos, a tradução do conhecimento e a assessoria técnico-científica para subsidiar decisões em saúde. No Brasil, a EVIPNet é coordenada pelo Ministério da Saúde, por meio do Departamento de Ciência e Tecnologia da Secretaria de Ciência, Tecnologia e Inovação em Saúde^8^.

Com o objetivo de reafirmar o compromisso com o fortalecimento e a disseminação das evidências, em parceria com a EVIPNet Brasil, a RESS abriu um chamamento especial para publicar pesquisas sobre políticas informadas por evidências, no segundo semestre de 2025. A iniciativa buscou reunir e divulgar artigos originais e revisões produzidos por pesquisadores sobre temas de interesse da saúde coletiva. Esses estudos apontaram métodos de síntese de evidências comuns na área de políticas informadas por evidências e, como resultado, foram publicadas seis revisões e três artigos originais, os quais fornecem subsídios para a tomada de decisão em políticas públicas de saúde e constituem o objeto deste editorial. 

Um levantamento realizado entre 2024 e 2025 sobre os Núcleos de Evidência (NEv) do Brasil observou que, apesar da disponibilidade de infraestrutura básica e do acesso a bases científicas, persistem limitações relacionadas à escassez de recursos financeiros e de pessoal qualificado^9^. De forma semelhante, a avaliação da capacidade institucional dos cinco departamentos da Secretaria de Ciência, Tecnologia e Inovação em Saúde identificou, em 2024, elevada qualificação dos profissionais e conhecimento sobre políticas informadas por evidências, mas também desigualdades entre departamentos e barreiras ao uso de evidências, como restrição de tempo, dificuldades de acesso a artigos científicos e ausência de ferramentas de tradução do conhecimento^10^. Complementarmente, uma revisão de escopo observou que os programas de residência em saúde contribuem para o fortalecimento do capital humano, qualificando a prática profissional e favorecendo a integração entre ensino e serviço no Brasil^11^.

Uma síntese de evidências apresentou estratégias para enfrentar a hesitação vacinal e identificou opções, como o fortalecimento da comunicação, a implementação de programas de educação para profissionais de saúde, a ampliação do acesso da população à vacinação e a integração de estratégias de vacinação para gestantes^12^. Uma revisão rápida de revisões sistemáticas mostrou que o uso de inseticidas, a notificação comunitária, o monitoramento de animais domésticos e silvestres, bem como as ações educativas e de manejo, seriam importantes para controlar e prevenir a doença de Chagas^13^. Essas sínteses podem apoiar a implementação de programas e ações no contexto do SUS.

Outra revisão de revisões sistemáticas contemplou a promoção da saúde sexual e reprodutiva de adolescentes e jovens adultos no Brasil. Observou-se que a maioria das intervenções ocorreu em ambiente escolar e teve como pauta o uso de anticoncepcionais. Além disso, apontou-se a relevância de se adotar uma abordagem multidisciplinar na promoção da saúde^14^. Um estudo transversal, realizado com escolares de Cubatão/SP, evidenciou que a exposição à poluição atmosférica foi associada à redução da variabilidade da frequência cardíaca e da função pulmonar^15^. 

A influência das desigualdades em aspectos da assistência e da geração de evidências foi discutida em diferentes artigos. Uma revisão de escopo revelou que a maioria dos estudos explorou a natureza biológica das deficiências, mas poucos abordaram marcadores político-sociais, reforçando que há lacunas no enfrentamento desse tema no Brasil^16^. Uma revisão sistemática identificou que as hospitalizações e a mortalidade por covid-19 provavelmente foram impulsionadas por desigualdades socioeconômicas e pelo acesso desigual à saúde no Brasil, com impactos maiores nas pessoas da raça/cor negra^17^. Essas contribuições consideraram a realidade brasileira e favorecem a implantação de políticas informadas por evidências nas áreas analisadas.

Os estudos que atenderam ao chamamento da RESS, motivado pela Rede EVIPNet Brasil, sistematizaram evidências globais e locais que fornecem subsídios para a tomada de decisão em políticas públicas de saúde. A inclusão de temas e métodos diversos revela a maturidade das pesquisas incentivadas no contexto da EVIPNet Brasil na produção de evidências para a tomada de decisão. Ao divulgar os resultados dessas pesquisas, a RESS reafirma seu papel na disseminação de evidências que contribuem para o aprimoramento do SUS.
